# Differential Signature of the Microbiome and Neutrophils in the Oral Cavity of HIV-Infected Individuals

**DOI:** 10.3389/fimmu.2021.780910

**Published:** 2021-11-09

**Authors:** Eliana Perez Rosero, Samantha Heron, Juan Jovel, Conar R. O’Neil, Shannon Lee Turvey, Pallavi Parashar, Shokrollah Elahi

**Affiliations:** ^1^ Department of Dentistry, University of Alberta, Edmonton, AB, Canada; ^2^ Department of Medicine, Division of Infectious Disease, University of Alberta, Edmonton, AB, Canada; ^3^ Department of Medical Microbiology and Immunology, University of Alberta, Edmonton, AB, Canada; ^4^ Department of Oncology, University of Alberta, Edmonton, AB, Canada; ^5^ Li Ka Shing Institute of Virology, Faculty of Medicine and Dentistry, University of Alberta, Edmonton, AB, Canada

**Keywords:** neutrophils, oral microbiome, HIV infection, galectin-9, CD44

## Abstract

HIV infection is associated with a wide range of changes in microbial communities and immune cell components of the oral cavity. The purpose of this study was to evaluate the oral microbiome in relationship to oral neutrophils in HIV-infected compared to healthy individuals. We evaluated oral washes and saliva samples from HIV-infected individuals (n=52) and healthy controls (n=43). Using 16S-rRNA gene sequencing, we found differential β-diversity using Principal Coordinate Analysis (PCoA) with Bray-Curtis distances. The α-diversity analysis by Faith’s, Shannon, and observed OTUs indexes indicated that the saliva samples from HIV-infected individuals harbored significantly richer bacterial communities compared to the saliva samples from healthy individuals. Notably, we observed that five species of Spirochaeta including *Spirochaetaceae*, *Spirochaeta*, *Treponema*, *Treponema amylovorum*, and *Treponema azotonutricum* were significantly abundant. In contrast, *Helicobacter* species were significantly reduced in the saliva of HIV-infected individuals. Moreover, we found a significant reduction in the frequency of oral neutrophils in the oral cavity of HIV-infected individuals, which was positively related to their CD4^+^ T cell count. In particular, we noted a significant decline in CD44 expressing neutrophils and the intensity of CD44 expression on oral neutrophils of HIV-infected individuals. This observation was supported by the elevation of soluble CD44 in the saliva of HIV-infected individuals. Overall, the core oral microbiome was distinguishable between HIV-infected individuals on antiretroviral therapy compared to the HIV-negative group. The observed reduction in oral neutrophils might likely be related to the low surface expression of CD44, resulting in a higher bacterial diversity and richness in HIV-infected individuals.

## Introduction

The oral cavity is a unique environment that comprises immune cells, soluble immune mediators, microbial communities, food antigens/foreign materials, soft and hard tissues (1, 2). Although most of the research has been focused on bacterial communities, the oral cavity harbors a large collection of viruses, fungi, and bacteriophages (3). Saliva flow, soluble salivary components such as antimicrobial peptides (4), immune cells, and mucosal epithelial cells in cross-talk with oral microbiome work together to sustain an immune homeostatic state under normal physiological conditions (5, 6). For example, SLPI (salivary secretory leukocyte protease inhibitor), defensins, sIgA, lactoferrin, and lysosome in the saliva play a protective role against viral infections (e.g. HIV) (7). However, upon the acquisition of HIV infection and disease progression due to the elimination/reduction of HIV-target cells (CD4^+^ and Th17 cells) (7–9), patients become prone to opportunistic infections such as candidiasis and other oral manifestations (e.g. Kaposi’s sarcoma) (10, 11). Notably, elimination of Th17 cells and reduction in IL-17 result in decreased recruitment of innate immune cells especially neutrophils into the oral cavity (12). Besides, HIV-infected individuals with lower CD4^+^ T cell count even when on antiretroviral therapy (ART) exhibit decreased neutrophil frequency in their blood circulation (13).

Neutrophils are the most abundant leukocytes in the blood circulation and also in the oral cavity (14). The interaction of neutrophils with symbiotic microbial communities plays a crucial role in immune homeostasis at the mucosal surfaces such as the oral cavity (15). However, dysbiotic microbiota at the mucosal surfaces (e.g. gingival crevice) activate neutrophils which results in an exacerbated inflammatory response (16). The oral cavity is an important peripheral microenvironment for neutrophils given its richness with bacterial communities (17). Additionally, bacterial communities can directly or indirectly (e.g. activation of macrophage, dendritic cells, and lymphocytes) influence neutrophil recruitment and activation in oral tissues (15, 18). For example, germ-free mice have decreased neutrophils and their progenitors in the oral cavity compared to wild-type mice (19). It has also been reported that bacterial communities enhance neutrophils aging (20). As such, aged neutrophils exhibit an inflammatory phenotype by increased production of reactive oxygen species (ROS) (21). Moreover, chronic conditions can influence microbial communities at the mucosal surfaces including the oral cavity. For example, diabetes can modify the oral microbiome to exacerbate periodontal disease (22).On the other hand, the dysbiotic oral microbiome has been reported to be associated with colorectal and pancreatic cancers (23–25). Nevertheless, the impact of HIV infection on the oral microbiome is controversial. Some studies support microbiome modifications, and some do not. These discrepancies could be related to various factors such as the study design, detection methods, and sampling (e.g., saliva, oral wash, or subgingival). For example, it was reported that HIV infection modulates oral fungi population and cigarette smoking in HIV-infected individuals was associated with greater microbial diversity (26). It appears that HIV infection shifts oral microbial communities towards a dysbiotic state (27). In particular, higher levels of cultivable microbes were isolated from the saliva of HIV-infected individuals compared to the HIV-uninfected group (28, 29). Other clinical variables are associated with alterations in the composition of oral microbial communities in HIV-infected individuals on ART (29). Although the potential influence of ART on the oral microbiome is debatable, it is reported that ART partially reverses HIV-induced oral microbiota alteration (29). ART treatment appeared to be associated with significantly greater bacterial richness and diversity (30). These studies demonstrate that HIV infection and/or ART adds another layer of complexity to the tight interplay between the immune system and microbial communities in the oral cavity. However, the impact of HIV infection and/or microbial communities on oral neutrophils and vice versa have remained unexplored.

Neutrophils are crucial players in immune homeostasis in the oral cavity (31). However, in inflammatory conditions such as periodontitis, the accumulation of activated neutrophils can result in tissue damage and bone loss (32). The activation of circulatory neutrophils results in cellular polarization, which facilitates tissue extravasation (33). CD44, a type I transmembrane glycoprotein, is one of the extracellular adhesion molecules that impacts neutrophil rolling and tissue migration (34). As such, lack of CD44 was associated with decreased neutrophil migration to inflamed tissue in CD44 KO mice (35). Besides, the interaction of CD44 with hyaluronate enhances neutrophil phagocytosis *in vitro* (36). Recently, we reported that CD44 interacts with Galectin-9 (Gal-9) on blood neutrophils (37). Gal-9 as a beta-galactosidase binding protein has a wide range of immunomodulatory properties depending on interaction with its corresponding receptors (38). We found that CD44 depalmitoylate during neutrophil activation and facilitates the movement of CD44 out of the lipid raft, and subsequently Gal-9 shedding from neutrophils in HIV-infected individuals (37). This process results in increased soluble plasma Gal-9 in HIV-infected individuals which subsequently enhances T cell activation *via* interaction with CD44 on T cells (37). Therefore, Gal-9 shedding from neutrophils might explain a potential source for the elevated plasma Gal-9 in HIV-infected individuals (39).

In the present study, we show that HIV-infected individuals have a different and richer bacterial composition in their saliva than healthy controls. Besides, we observed a significant reduction in the proportion of oral neutrophils in HIV-infected individuals, in particular, in those with lower CD4^+^ T cell count. Additionally, we observed downregulation of CD44 surface expression on oral neutrophils in HIV-infected individuals, which potentially explains their decreased frequency in the oral cavity of HIV infected-individuals.

## Materials and Methods

### Study Population

For this study, we recruited sixty-one HIV-infected individuals including: a) on ART with low CD4^+^ T cell count (< 200 cells/mm^3^, n=11); b) on ART with high CD4^+^ count (>200 cells/mm^3^, n=40); c) Long-term non-progressors (40) (LTNPs, n=9) and d) ART-native, n=2) through the Northern Alberta HIV Program in Edmonton, Canada (**Supplementary Tables 1, 2**). All the work presented in this manuscript was conducted on HIV-infected individuals receiving ART except **Figures 2C, D**, which compares the frequency of neutrophils in different HIV-infected groups. Also, a total of 43 healthy controls (HCs) defined as HIV, Hepatitis B virus, and Hepatitis C virus seronegative individuals without active oral disease were recruited at the University of Alberta for comparison. The institutional ethics review boards at the University of Alberta approved the study with the protocols (Pro00070528 and Pro000064046). All study participants gave written informed consent to participate in the study.

### Sample Collection

Participants avoided eating or drinking for at least 30 minutes before the sample collection. Saliva samples were obtained followed by oral washes from the study participants. Saliva samples were aliquoted and stored at -80°C until use.

Oral wash was performed 5 times using 15 ml of phosphate-buffered saline solution (PBS) for 30 seconds with 3 minutes’ intermission between rinses. Samples were centrifuged at 2000 rpm for 10 min, supernatants were discarded, and cell pellets were resuspended in culture media (RPMI-1640) supplemented with 10% fetal bovine serum (FBS) (Sigma) and 1% penicillin-streptomycin (Sigma). Cell suspensions were filtered through 100, 70, and 50 μm sterile strainers (Fischer Scientific), centrifuged and resuspended in culture media for further analysis. Blood samples of 16 HCs and 15 HIV-infected individuals on ART were subjected to gradient separation using Ficoll-Paque Premium (GE). The peripheral blood mononuclear cell (PBMC) fraction was removed, and the remaining red blood cell pellet was lysed using red blood cell lysis buffer for 10 minutes (0.155M NH_4_Cl, 10mM KHCO_3_, and 0.1mM EDTA) to isolate polymorphonuclear cells according to our previous methods (37, 41).

### Flow Cytometry Analysis

Fluorophore antibodies with specificity to antigens of human cells were purchased from BD Biosciences, Thermo Fisher Scientific, and/or R&D. We used anti-CD15 (clone W6D3, Cat#555402), anti-Gal-9 (clone 9M1-3, Cat#50-9116-42), anti-CD44 (clone 515, Cat#562890), anti-CD16 (clone 3G8, Cat#557744) and anti-CD32 (clone FLI8.26, Cat#744259). Cell viability was evaluated by LIVE/DEAD Kit (ThermoFisher Scientific Cat#L34966). Apoptosis assay was performed using the PE Annexin V Apoptosis Detection Kit I (BD Biosciences Cat#55963) according to the manufacturer’s protocol. Stained cells were fixed in 4% paraformaldehyde before acquiring on an LSRFortessa-SORP or LSRFortessa X-20 flow cytometers (BD Biosciences), and data were analyzed using the FlowJo (version 10).

### Cytokine, CD44, and Gal-9 Measurement

The liquid fraction of the saliva was 2-fold diluted for cytokine quantification. We specifically measured TNF-α, IL-8, IL-6, IL-10, IL-13, IL-1B, IFN-γ using V-plex Plus pro-inflammatory kit from Meso Scale Discovery (MSD Cat#K15054D-1), according to the manufacturer’s instruction and our previous reports (41, 42). Similarly, CD44 (R&D Systems; DY7045-05) and Gal-9 (R&D; DY 2045) concentrations were quantified by ELISA.

### Neutrophil Migration Assay

Chemotaxis was evaluated by the Cell Invasion Assay (Millipore Sigma, Cat. ECM 555). Blood neutrophils (n=10) and oral neutrophils (n=6) from HCs (n=10) were isolated and 0.5 million cells were used per well. Cells were pre-incubated in a 96 well plate with the anti-CD44 antibody (TermoFisher, Cat. 16-0441-85) at 37°C, for 30 min. Then cells were transported to the upper chamber of the migration assay and incubated in the presence or absence of soluble Galectin-9 (1.5 ng/ml) for 3 hours at 37°C. N-Formylmethionyl-leucyl-phenylalanine fMLP (50 μm, Sigma Aldrich, Cat. F3506-10MG) was used as a chemoattractant in the lower chamber. Culture media was used as the negative control. The migrated cells were lyzed and quantified by measuring the relative fluorescence units per manufacturing protocol using the plate reader a5 480/520 nm.

### Bacterial DNA Isolation

Saliva aliquots from sex and age paired participants were centrifuged and pellets were used for DNA isolation using the QIAamp DNA Mini Kit (Cat# 51304). Pellets were mixed with 20 μl of Proteinase K and 200 μl of in-house lysis buffer (100 mL of 0.5 M sodium chloride, 0.005 M tris aminomethane-pH8, 0.05M ethylenediaminetetraacetic acid with pH 8, and 4% sodium dodecyl sulfate), briefly vortexed, and incubated at 56°C water bath for 60 min, followed by 15 min incubation at 70°C. Then 200 μl of buffer AL (from the kit) was added for a final 10-minute incubation at 70°C, accompanied by DNA column extraction according to the manufacturer’s instructions.

### 16SrRNA Illumina MiSeq Sequencing

V3-V4 variable regions of 16S rRNA were amplified from genomic DNA samples. Amplicons were generated using the following primers: Forward Primer = 5’ CGTCGGCAGCGTCAGATGTGTATAAGAGACAGCCTACGGGNGGCWGCAG and Reverse Primer = 5’ TCTCGTGGGCTCGGAGATGTGTATAAGAGACAGGACTAC HVGGGTATCTAATCC (IDT). Thus, generated PCR products were indexed using Illumina’s Nextera XT kit. Sequencing was performed on Illumina’s MiSeq platform using a 250-bp paired-end sequencing kit at The Applied Genomic Core (TAGC), University of Alberta.

Sequencing data were demultiplexed and binned into individual samples according to their barcodes and further bioinformatic analysis was performed using the QIIME2 pipeline (2021.4). The first step of this analysis was to join the paired-end reads (1 and 2) with a minimum of 100 bp overlap and 0 mismatches. Reads were then quality filtered by removing sequences having more than 10 sites with a Phred quality score less than 20. Next, reads were denoised into amplicon sequence variants (ASVs) using the DADA method. Taxonomy classification at the phylum, family, and genus levels was done by comparing ASVs to the Green genes bacterial reference database (v. 13.5). Diversity indices (Evenness, Observed OTUs, Shannon’s diversity, and Faith’s phylogenetic index) and distances between samples (Bray-Curtis, weighted- and unweighted-Unifrac) were all calculated in QIIME2 to profile oral microbiota. Before taxonomy classification and generating alpha and beta diversity metrics, data were rarefied across samples for normalization such that all samples have the same number of total reads.

### Statistical Analysis

This was performed in GraphPad Prism 9 (GraphPad Software, Inc.). D’Agostino & Pearson test was used for normality check, being samples non-parametrical Mann-Whitney U tests for unpaired data or Wilcoxon signed-rank tests for paired data were used. When comparing more than two groups, the Kruskal Wallis test was used. Means and standard deviations (mean ± SD) are used to present the data. Correlation analysis was performed by nonparametric Spearman correlation.

## Results

### Differential Bacterial Communities in the Oral Cavity of HIV-Infected Individuals Compared to HCs

**Figure 1:**
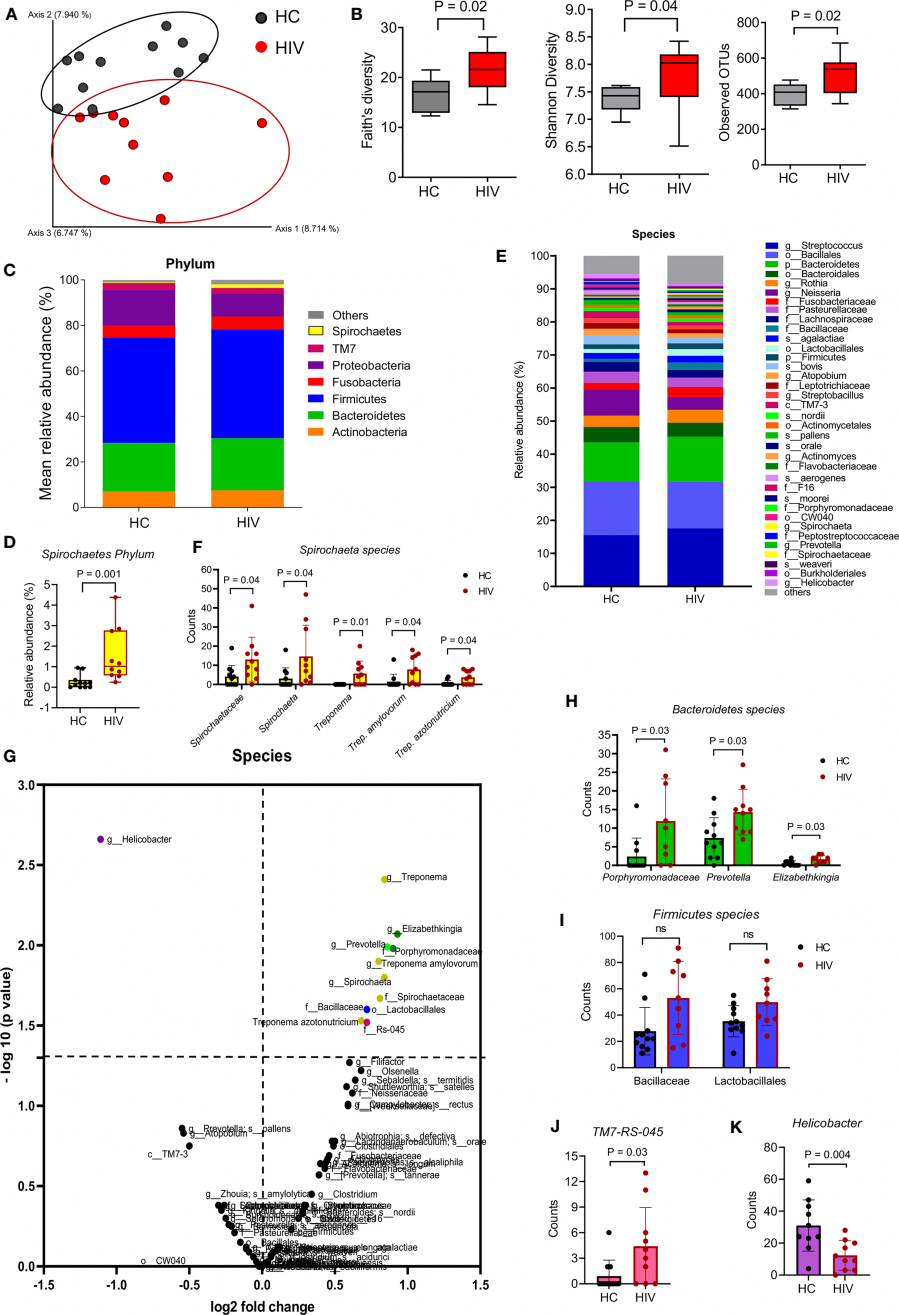
Differential oral microbiome in the saliva of HIV-infected compared to healthy individuals. **(A)** Beta diversity in bacterial communities is shown by Principal coordinates analysis (PCOA) of Bray-Curtis Distances, in gray HCs (n = 11), in red HIV-infected individuals (n = 10), and each symbol represents an individual. **(B)** Alpha diversity in bacterial communities determine by Faith’s, Shannon’s, and observed OTUs indexes. **(C)** Comparison of the saliva microbiota at phylum level in HCs versus HIV-infected group. **(D)** Comparison of *Spirochaete’s* phylum in the saliva of HCs versus HIV-infected individuals. **(E)** Comparison of the saliva microbiota at the species level in HCs versus HIV-infected group. Approximately, 32% of all taxa resolved could be classified at the species level. All other taxa are also included in the barplot at the lowest level they could be resolved (see legend: s_: species, g_: genus, f_: family, o_: order, c_ class, p_: phylum). **(F)** Comparison of unclassified *Spirochaetaceae, Spirochaeta, Treponema, Trep. amylovorum*, and *Trep. Azotonutricum* from *Spirochaete’s* phylum in the saliva of HCs versus HIV-infected individuals. **(G)** The volcano plot comparing bacterial Genus in the saliva samples from HCs versus HIV-infected individuals. All genus that exceeded the dotted line marked at 1.3 were considered significant. **(H)** Comparison of *Bacteroidetes* genus members *Prophyromonadaceae, Prevotella, and Elizabethkingia* in saliva samples of HCs versus HIV-infected individuals. **(I)** Comparison of *Firmicutes* genus members *Bacillaceae and Lactobacillales* in saliva samples of HCs versus HIV-infected individuals. **(J)** Comparison of *Sebaldella* genus from *Fusobacteria* phyla (TM7-RS=045) in saliva samples of HCs versus HIV-infected individuals. **(K)** Comparison of *Helicobacter* genus in saliva samples of HCs versus HIV-infected individuals. ns, no significant.

We first compared the two groups in terms of β-diversity using Principal Coordinate Analysis (PCoA) with Bray-Curtis distances. The results indicated a differential clustering of bacterial communities in the saliva of HIV-infected compared to healthy individuals (**Figure 1A**). Distances within groups versus distances across groups were determined by ANOSIM, which was significant (P=0.001). ADONIS or differences between the group’s centroid was also significant (P=0.003). The Alpha diversity analysis by Faith’s, Shannon, and observed OTUs indexes showed that the saliva samples from HIV-infected individuals harbored significantly richer bacterial communities compared to the saliva samples from HCs (**Figure 1B**)

To further determine which bacterial communities were different between the groups, we compared them at different levels. At the phylum level, we found Spirochaetes’ phylum was significantly enriched in the saliva samples of HIV-infected individuals compared to HCs (**Figures 1C, D**). When bacterial communities were compared at the species level (**Figure 1E**), we observed that five species of Spirochaeta including *Spirochaetaceae*, *Spirochaeta*, *Treponema*, Treponema *amylovorum*, and *Treponema azotonutricum* were significantly abundant in the saliva of HIV-infected individuals compared to HCs (**Figure 1F**). The volcano plot shows further differences in bacterial species among the groups. Species that were enriched in the saliva of HIV-infected individuals are shown in the right upper quadrant, and bacterial species that were less prevalent in the saliva of HIV-infected individuals are shown in the upper left quadrant of the volcano plot (**Figure 1G**). Bacterial species with mean counts less than 1 were not accounted for. We found that bacterial communities belonging to *Spirochaetes*, *Bacteroidetes*, *Firmicutes*, and *TM7* species were significantly enriched in the saliva samples from HIV-infected individuals, respectively (**Figures 1H–J**). In contrast, we noted a significantly lower abundance of Proteobacteria phylum (in particular *Helicobacter*) in the saliva of HIV-infected individuals compared to HCs (**Figure 1K**). We used 97% sequence identity for binning analysis. Overall, our results show a significant difference in bacterial communities in the oral cavity of HIV-infected compared to uninfected individuals. To exclude potential effects of sex/age, saliva specimens were age-sex-matched for the microbiome studies.

### A Lower Proportion of Neutrophils in the Oral Cavity Is Related to the Clinical Status of HIV-Infected Individuals

**Figure 2:**
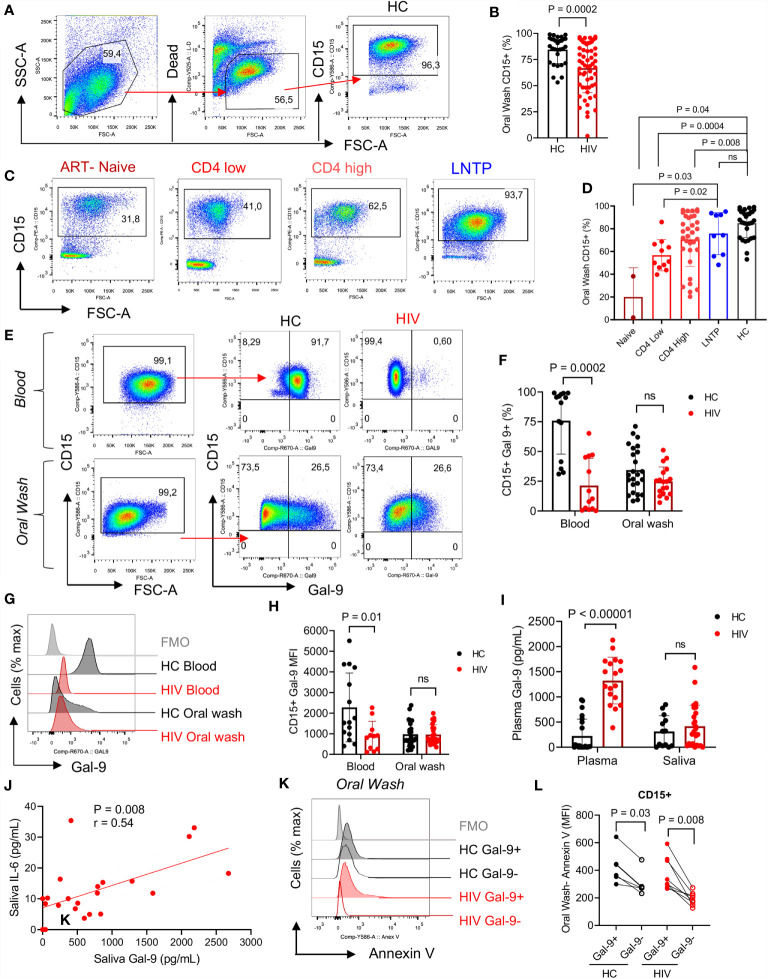
Neutrophils in the oral cavity are decreased in HIV-infected individuals and have low surface Gal-9. **(A)** Representative flow cytometry plots for CD15^+^ neutrophil identification in the oral wash. **(B)** Cumulative data of percentages of CD15^+^ cells in the oral washes of HCs compared to HIV-infected individuals. **(C)** Representative flow cytometry plots, and **(D)** cumulative data of neutrophils in oral washes of different HIV-infected individuals (ART-naïve, on ART with low CD4^+^ T cell count (< 200 cells/ul) or high CD4^+^ T cell count (> 200 cells/ul), and long-term non-progressor (LTNP) compared to HCs. **(E)** Representative flow cytometry plots, and **(F)** cumulative data of Gal-9 surface expression in neutrophils from the blood and oral washes of HIV and healthy individuals. **(G)** Representative histogram plots, and **(H)** cumulative data of Gal-9 expression measured by the Mean Fluorescence Intensity (MFI) on blood and oral neutrophils of HIV-infected and healthy individuals. **(I)** Cumulative data of soluble Gal-9 in the plasma and saliva of HIV-infected and healthy individuals as measured by ELISA. **(J)** Cumulative data of a positive correlation between the saliva Gal-9 with the saliva IL-6 (Spearman correlation, r = 0.54, P = 0.008). **(K)** Representative histogram plots, and **(L)** cumulative data of apoptosis in oral neutrophils in regard to Gal-9 expression measured by Annexin V assay. ns, no significant.

To better understand the possible role of immune components of the oral cavity in HIV-infected individuals on the bacterial composition, we focused on neutrophils as the most abundant immune cells in the oral cavity (43). In agreement with previous reports, we found that neutrophils were the most abundant cells in the oral cavity. However, HIV-infected individuals had significantly lower percentages of neutrophils in their oral cavity compared to HCs (**Figures 2A, B**). Both oral and blood neutrophils co-express CD15 and CD16 (**Supplementary Figures 1A, B**). Notably, we observed significantly lower percentages of neutrophils in the oral cavity of ART-naive and patients on ART compared to HCs (**Figures 2C, D**). In contrast, we found a similar proportion of oral neutrophils in the oral cavity of HIV-infected LTNPs compared to HCs (**Figures 2C, D**). These observations suggest that the disease status may impact the frequency of neutrophils at the mucosal surfaces. Moreover, we did not find any significant difference in the frequency of other immune cells (e.g., dendritic cells, monocytes, and B cells) in the oral cavity of HIV-infected versus HCs except CD4^+^ T cells. Although, in general, the percentage of T cells was very low (a few %), we found significantly lower percentages of CD4^+^ T cells but not CD8^+^ T cells in the oral cavity of HIV-infected individuals compared to HCs (**Supplementary Figures 1C–G**).

### Gal-9 Is Downregulated on the Surface of Blood but Not Oral Neutrophils in HIV-Infected Individuals

Recently, we showed that Gal-9 is downregulated from the surface of blood neutrophils in HIV-infected individuals compared to HCs (37). Therefore, we decided to investigate whether this was the case for oral neutrophils. Our observations reconfirmed that the frequency of Gal-9 expressing blood neutrophils was significantly lower in HIV-infected individuals compared to HCs (**Figures 2E, F**). However, we found a smaller portion of Gal-9 expressing oral neutrophils compared to their siblings in the blood, without any significant difference between the HIV-infected individuals on ART and HCs (**Figures 2E, F**). Moreover, we measured the intensity of Gal-9 and found that the Mean fluorescence intensity (MFI) of Gal-9 was significantly lower on the surface of blood neutrophils from HIV-infected individuals (**Figures 2G, H**). However, this was not the case for the oral neutrophils (**Figures 2G, H**). We also quantified the concentration of soluble Gal-9 in the plasma and saliva samples. Although soluble Gal-9 was significantly elevated in the plasma of HIV-infected individuals as we have reported elsewhere (39), we did not find any difference in the soluble Gal-9 levels in the saliva between the groups (**Figure 2I**).

### Soluble Gal-9 Is Positively Correlated With IL-6 in the Saliva of HIV-Infected Individuals

Since Gal-9 has been reported as a contributing factor to the cytokine storm in COVID-19 patients (41), we reasoned to evaluate the correlation of saliva Gal-9 levels with pro-inflammatory cytokines. We found that Gal-9 levels were positively correlated with IL-6 in the saliva (**Figure 2J**). This may suggest the potential role of soluble Gal-9 in the activation status of neutrophils in the oral cavity of HIV-infected individuals. However, this was not the case for other quantified pro-inflammatory cytokines (e.g., IL-1β, IL-8, TNF-α and IFN-γ) in the saliva.

### Downregulation of Surface Gal-9 Makes Oral Neutrophils Less Apoptotic

IL-6 has been related to increased neutrophil survival (44) and an activated status can prolong the lifespan of neutrophils (45). Thus, we decided to investigate differences in Gal-9+ versus Gal-9- oral neutrophils. Recently, we have reported that stimulation of blood neutrophils with LPS results in the downregulation of Gal-9 at the gene and protein levels (37). Thus, we proposed that activated neutrophils lose their surface Gal-9, which in turn increases their lifespan. Indeed, we found that in HCs and HIV-infected individuals, neutrophils that did have surface Gal-9 were less apoptotic compared to their Gal-9 expressing counterparts (**Figures 2K, L**). These observations suggest that the downregulation of Gal-9 may act as a mechanism of enhanced neutrophil survival.

### CD44 Is Downregulated From the Surface of Oral Neutrophils in HIV-Infected Individuals

**Figure 3:**
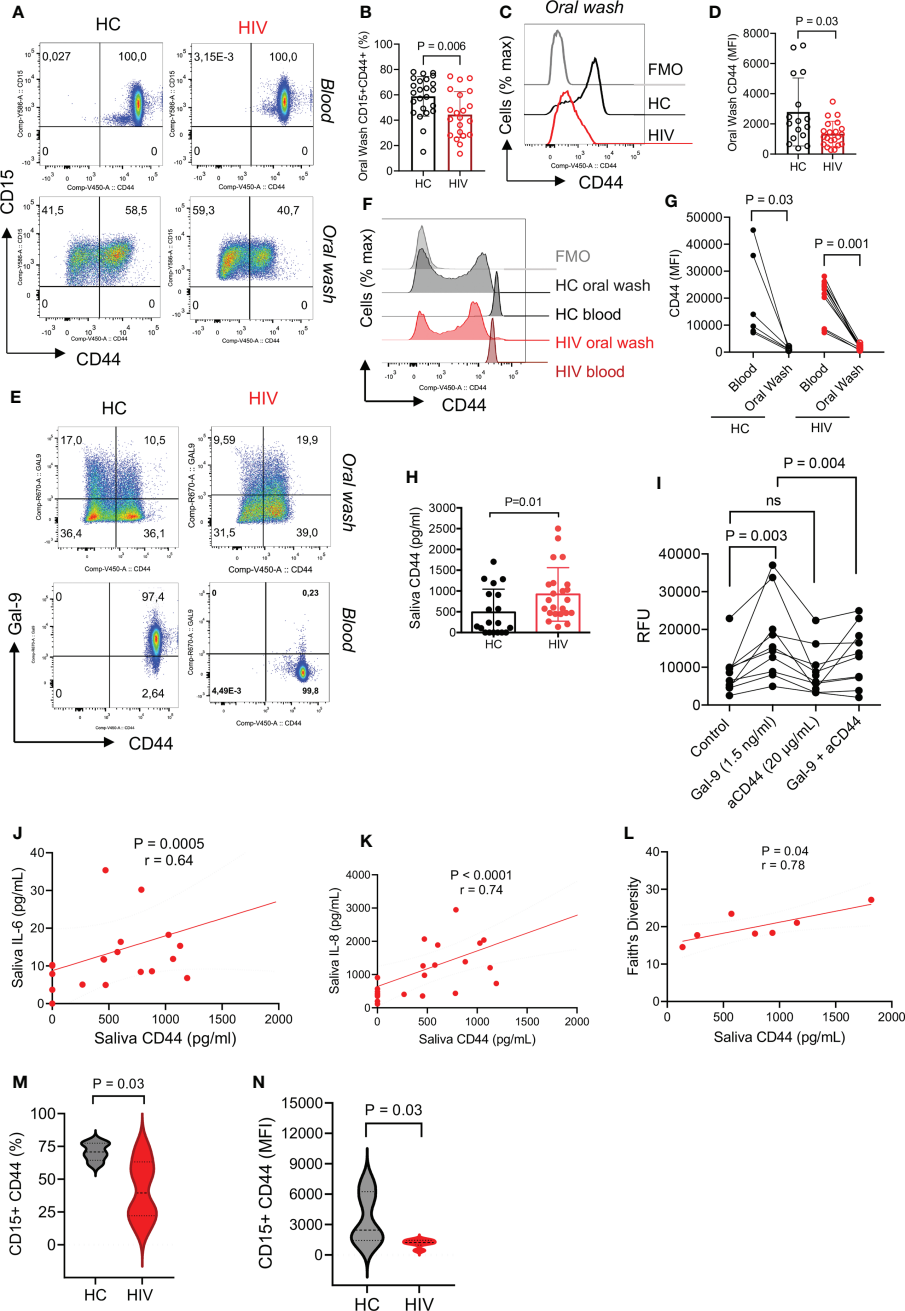
CD44 is downregulated from the surface of oral neutrophils in HIV-infected individuals. **(A)** Representative flow cytometry plots of CD45 expression on blood and oral neutrophils. **(B)** Cumulative data of CD45 expression on oral neutrophils of HIV-infected versus healthy individuals. **(C)** Representative histogram plots, and **(D)** cumulative data of CD44 expression (MFI) in oral neutrophils of HV-infected individuals vs. HCs. **(E)** Representative flow cytometry plots of co-expression of CD44 and Gal-9 in oral and blood neutrophils in HIV-infected vs. healthy individuals. **(F)** Representative histogram plots, and **(G)** cumulative data of CD44 expression (MFI) in the blood and oral neutrophils of HIV-infected vs. healthy individuals. **(H)** Concentrations of soluble CD44 in the saliva of HIV-infected vs. healthy individuals as measured by ELISA. **(I)** Cumulative data showing blood neutrophil migration in the presence of soluble Gal-9 (1.5 ng/ml), anti-CD44 antibody (20 μg/ml), or soluble Gal-9 (1.5 ng/ml) plus anti-CD44 antibody while all wells were treated with fMLP (50 μm). **(J)** Cumulative results showing a positive correlation between the soluble CD44 with IL-6 in the saliva of HIV-infected individuals, calculated by Spearman correlation **(K)** Cumulative results showing a positive correlation between the soluble CD44 with IL-8 in the saliva of HIV-infected individuals, calculated by Spearman correlation. **(L)** Cumulative data showing a positive correlation between the soluble CD44 with bacterial Faith’s diversity, measured by Spearman correlation. **(M)** Cumulative data of percentages of neutrophils expression CD44 in the oral cavity of HCs versus HIV-infected individuals that were subjected to 16S gene sequencing. **(N)** Cumulative data of the intensity of CD44 expression on neutrophils from the oral cavity of HCs versus HIV-infected individuals that were subjected to 16S gene sequencing. ns, no significant.

Unstimulated blood neutrophils express high levels of surface Gal-9 which is bound to CD44 (37). Therefore, we decided to determine whether the same pattern exists for oral neutrophils. Similar to our previous finding, we found all blood neutrophils expressed CD44, however, this was about 50% for oral neutrophils (**Figure 3A**). Interestingly, we observed that the percentage of CD44 expressing neutrophils was significantly lower in the oral cavity of HIV-infected individuals (**Figures 3A, B**). Moreover, we found a significant reduction in the intensity of CD44 expression on oral neutrophils from HIV-infected individuals versus HCs (**Figures 3C, D**). When the expression of CD44 in the blood and oral neutrophils was analyzed, we found a lower frequency of CD44 expressing neutrophils and even CD44 expression level on oral neutrophils compared to their counterparts in the blood in both HCs and HIV-infected individuals (**Figures 3E–G**). These observations led us to measure the soluble CD44 concentration in the saliva, which showed a significant elevation of CD44 levels in the saliva of HIV-infected compared to healthy individuals (**Figure 3H**). This might explain that oral neutrophils in HIV-infected individuals shed CD44 that can be detected in their saliva. Moreover, we found that the soluble Gal-9 promotes the migration of blood neutrophils *in vitro* (**Figure 3I**). In particular, we observed that this effect was CD44 dependent as the pre-incubation of neutrophils with the anti-CD44 antibody abrogated this effect (**Figure 3I**). Although a similar trend was observed for Gal-9 on neutrophils from the oral cavity, this effect did not reach a significant level (**Supplementary Figure 1H**). Therefore, these findings may suggest that CD44 in the blood is upregulated to facilitate neutrophil migration into peripheral tissues, but once they reach their action site it is downregulated to keep neutrophils in their destination.

### Soluble CD44 Is Associated With Inflammatory Cytokines and Bacterial Diversity

To further understand the possible role of soluble CD44 in the oral cavity, we found that it was positively correlated with saliva pro-inflammatory cytokines IL-6 and IL-8 in HIV-infected individuals (**Figures 3J, K**). Remarkably, we noted a positive correlation between the soluble CD44 with Faith’s bacterial diversity index in HIV-infected individuals (**Figure 3L**). To further delineate the correlation between neutrophils and the oral microbiome, we measured the frequency of CD44 expressing neutrophils and the intensity of CD44 expression on oral neutrophils from HIV-infected versus HCs that were subjected to the microbiome analysis. We found that oral neutrophils had a significantly lower proportion and expression level of CD44 compared to their counterparts in HCs (**Figures 3M, N**). These observations suggest that microbial-derived molecules and/or other factors in the oral cavity may influence the expression of CD44 in HIV-infected individuals.

### CD32 Is Highly Expressed in Oral Neutrophils From HIV-Infected Individuals and in CD44^+^ Neutrophils

**Figure 4:**
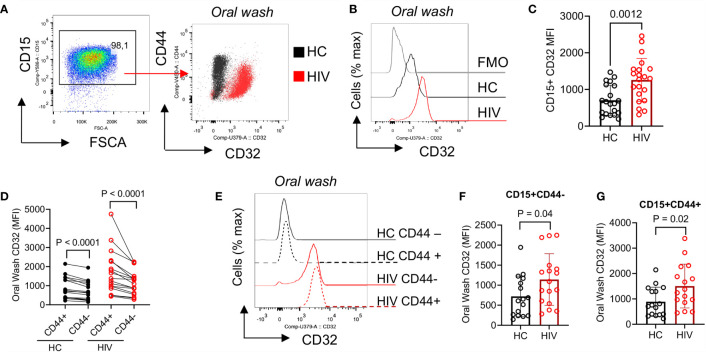
Differential expression of CD32 on neutrophils from HIV-infected individuals versus healthy individuals. **(A)** Representative flow cytometry plots of CD32 expression on neutrophils of an HIV-infected vs. a healthy individual. **(B)** Representative histogram plots, and **(C)** cumulative data of CD32 expression (MFI) in oral neutrophils from HIV-infected vs. healthy individuals. **(D)** Cumulative data of CD32 expression in regards to CD44 on neutrophils of HCs vs. HIV-infected individuals. flow cytometry plot of neutrophils from oral washes and their coexpression of CD44 and CD32 in HIV patients shown in red and HCs in black. Note the higher expression of CD32 in neutrophils of HIV patients. **(E)** Representative histogram plots, and **(F)** cumulative data of CD32 expression (MFI) in CD44^-^, and **(G)** CD44^+^ populations of oral neutrophils from HCs and HIV-infected individuals. Significance was calculated by the Kruskal Wallis test.

To further determine the activation status of neutrophils, we measured the expression of CD32. We found that CD32 was significantly upregulated in oral neutrophils of HIV-infected compared to healthy individuals (**Figures 4A–C**). CD32 has been described to participate in the interaction of neutrophils and uptake of IgG-opsonized viral particles (46). Thus, we decided to investigate whether aged oral neutrophils expressing CD44 had different CD32 expressions compared to CD44-negative counterparts. We found that CD44^+^ oral neutrophils had significantly a higher expression of CD32 compared to their CD44^-^ counterparts in both HIV-infected and healthy individuals (**Figure 4D**). In addition, we noted that although CD44^+^ neutrophils were more activated, both CD44^+^ and CD44^-^ neutrophils in HIV-infected individuals had significantly higher expression of CD32 compared to their counterparts in HCs (**Figures 4E–G**). These results suggest that neutrophils in the oral cavity of HIV-infected individuals have an activated phenotype compared to their counterparts in HCs.

### Patients’ Demographics and Oral Health Associated Factors

**Figure 5:**
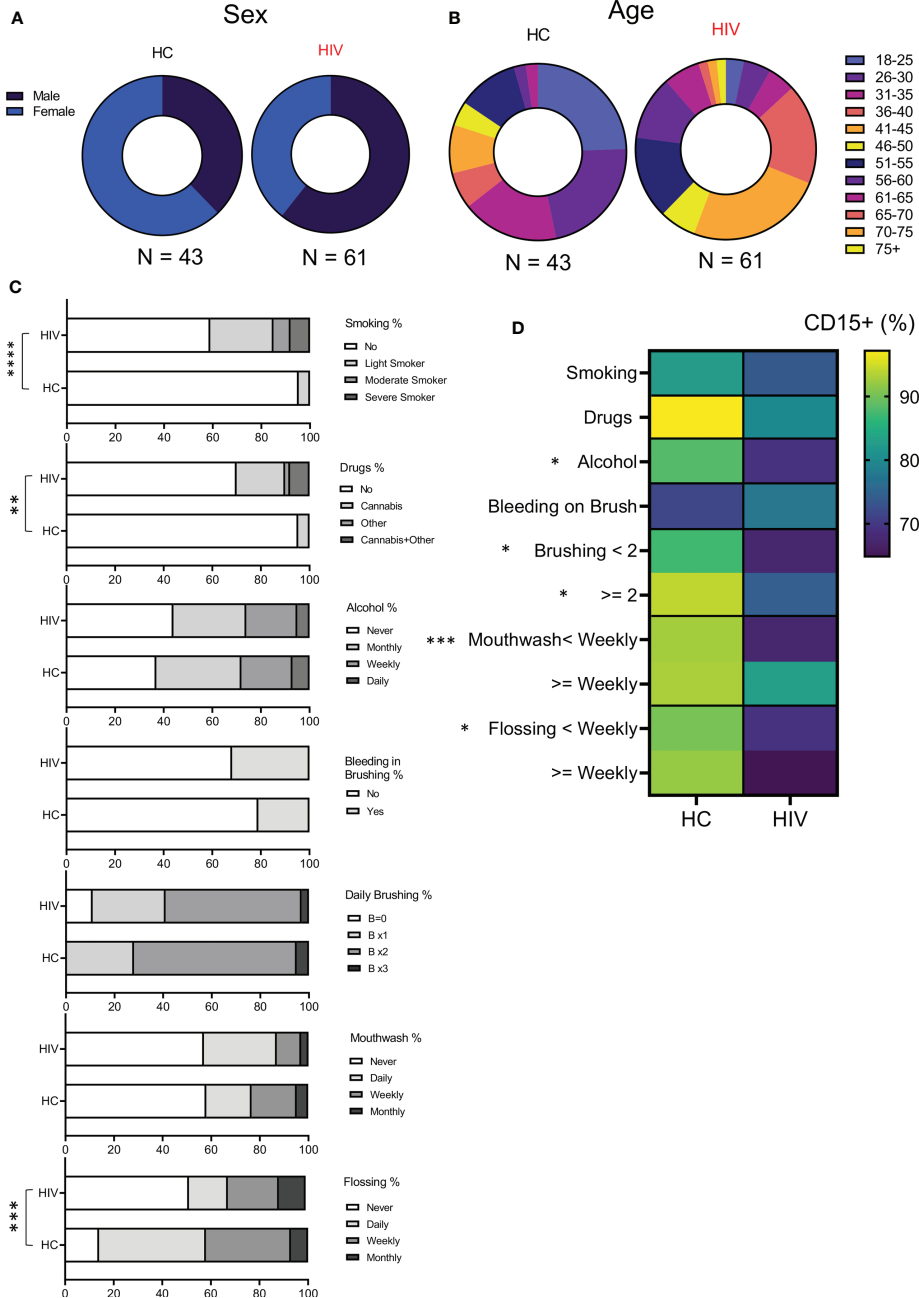
Demographics and habits assessment of participants. **(A)** Showing the sex distribution with 37% and 63% females and males, respectively in HCs, 61% and 39% for males and females in HIV-infected group. **(B)** Showing the age distribution among participants. HC group was enriched with younger population according to Fischer’s exact test analysis (P = 0.02). **(C)** Cumulative data illustrating participant’s habits such as smoking, drug use, alcohol consumption, daily oral health. **(D)** Cumulative data showing the correlation of neutrophils’ percentages with study participants’ habits. (****P < 0.0001, ***P = 0.0001, **P < 0.001, *P < 0.05).

To better understand whether observed changes were associated with other variables in both groups, we examined their sex, age, medications, oral health (brushing, flossing, using mouthwash), and other habits (smoking, alcohol, and recreational drug use) into our analysis (**Supplementary Table 3**). We found that groups were differently distributed based on their sex (P=0.028) (**Figure 5A**). While HCs consisted of 16 Males and 27 females, the HIV cohort was composed of 37 males and 24 females. In terms of age, for statistical analysis the Fischer exact test was applied that showed age was also different between groups (P= 0.027). HCs had more participants <50 years compared to HIV-infected individuals who were 50 < years old (**Figure 5B**). Excluding LTNPs and naïve patients, the rest of HIV-infected individuals were receiving ART. Also, some HIV-infected individuals (n=33) had other comorbidities (mainly diabetes, high cholesterol, and blood pressure, rheumatoid arthritis, anxiety-depression, and chronic pain). In contrast, a small portion of HIV-uninfected participants (n=13) had underlying conditions and were receiving related medications at the time of examination.

Also, the participant’s had differences in their habits as was analyzed by Fischer’s exact test. The HIV-infected group had a significantly higher number of smokers compared to HCs (P < 0.0001). The same pattern was true for recreational drug/cannabis in HIV-infected individuals compared to HCs (P=0.002) (**Figure 5C**). However, alcohol consumption was similar between the groups (**Figure 5C**). Regarding oral health, HIV-infected individuals flossed less often compared to HCs (P= 0.0001) (**Figure 5C**). In terms of brushing, mouthwash use, and the self-reported evaluation for bleeding on brushing (a tool for periodontitis risk prediction), we did not find any difference between the groups (**Figure 5C**). Finally, we correlated the proportion of neutrophils in oral washes (CD15^+^ cells) with habit practices in HIV-infected versus HCs. We found that HCs tend to have more neutrophils in their oral wash compared to HIV-infected individuals regardless of their habits (**Figure 5D**). Also, we noted that alcohol consumption, daily brushing, mouthwash use, and flossing significantly impacted neutrophil % in HCs vs HIV-infected individuals. Of note, the self-report for bleeding on brushing did not impact neutrophil percentages in the oral wash.

## Discussion

In this study, we investigated microbial communities in the saliva of HIV-infected and age-sex-matched HCs. We found differential bacterial compositions in the saliva of HIV-infected compared to healthy individuals. Our results are in agreement with another report that demonstrated differential bacterial clusters in the oral washes of a larger cohort of HIV-infected individuals versus HCs regardless of other contributing factors (e.g. smoking, missing teeth, gingivitis, candida infection, etc.) (29). We observed differences in bacterial α-diversity (e.g., Shannon and Faith’s indexes) and bacterial richness, which supports HIV-associated salivary dysbiosis. Although previous work investigated dysbiosis of the tongue (47) and periodontal tissues in HIV-infected individuals (48), our results support that saliva expectoration provides a non-invasive, less expensive, and informative approach for oral cavity microbiome-related studies. In addition to microbiome studies, our study bridges the correlation between HIV-immune status, oral soluble mediators, and oral neutrophils. In a previous work, major difference in the phyla of *Bacteroidetes*, *Firmicutes*, *Proteobacteria* and *TM7* in the saliva of HIV-infected individuals versus HCs has been documented (49). Our results were somewhat similar in identifying increases in oral *Spirochaetes, Bacteroidetes, Firmicutes*, and *TM7* in HIV-infected individuals. Such differential oral microbiome composition may be related to systemic inflammation and impaired pulmonary function in HIV-infected individuals as a contributing factor to chronic obstructive pulmonary disease (COPD) (50). Since all of our study subjects for the oral microbiome-related studies were on ART, we were unable to investigate the potential effects of ART on the oral microbiome. However, it was reported that the dysbiotic oral microbiome was not fully restored after effective ART, although some microbiota were restored (51). Furthermore, ART, viral load, and CD4^+^ T cell count differentially contribute to salivary dysbiosis resulting in reduced or increased different bacterial species (27).

It is difficult to differentiate between various factors without longitudinal studies pre-infection, pre-ART, the type of ART regimens, and well-matched HIV-infected untreated controls sampling. In general, oral health, habits such as smoking, and drinking may influence the oral microbial communities. Although in our cohort we did not observe any difference between the HIV-infected vs. the control group in terms of periodontitis, higher bacterial diversity and richness have been reported in HIV-infected individuals with severe periodontitis (52). Therefore, oral health can have direct effects on the microbial communities as increased bacterial diversity and richness have been associated with periodontal disease (53). Prior work also reported gingival bleeding, decayed teeth, periodontal pockets, and smoking habits as important contributing factor to oral dysbiosis (54). In our study, we observed that the HIV-infected group was more enriched with smokers, people who use recreational drug, and individuals who had poor adherence to oral health. These factors may influence the general oral health and subsequently the microbial composition of the oral cavity. For example, smoking has been associated with alteration in the oral microbiome. In particular, it has been reported that smoking enhances bacterial colonization in the upper respiratory tract in HCs, and even more pronounced in HIV-infected individuals (50, 55).

Although a greater abundance of *streptococcus* mutants, *lactobacillus*, and *candida* species has been in the saliva of HIV-infected individuals (47), we did not find such differences. Instead, we discovered significantly higher abundance of *Spirochaetes* (*Spirochaetacea*, *Spirochaeta, and Treponema), Porphyromonadaceae, Prevotella, Elizabethkingia, TM7* in the saliva of HIV-infected individuals. These observations are in agreement with a report showing an increased abundance of *Prevotella* in HIV-infected individuals (27). Similarly, an increase in *Porphyromonadaceae* in the saliva of HIV-infected individuals was reported at the start of ART when compared to 24 weeks later (30). Interestingly, the *Porphyromonadaceae family* harbors the well know periodontal pathogens *Phorphyromona gingivalis* and *Tannerella Forsythia* (56, 57). It is reported that *Phorphyromona gingivalis* partners with HIV-virus to co-infect mucosal epithelial cells, *in vitro* (58). Based on this observation, the abundance of *Phorphyormonas* in the oral cavity may facilitate HIV acquisition at the mucosal sites. However, further studies are required to support this hypothesis. *TM7-RS-045* or *Saccharibacteria* is a recently discovered commensal oral bacteria that abides at the expense of *Actynomices* bacteria, however, its role in the oral cavity is not well defined (59). In our cohort, we observed an increase of *TM7*, although we did not observe any difference in the abundance of *Actinomyces species*. A previous study, in support of our results, reported a higher abundance of *TM7, Treponema, and Prevotella* in the oral washes of HIV-infected individuals (29). Particularly, *Treponema denticola* associated with *Porphyromonas gingivalis, and Tanerella forsythia* form the “red complex”, which are the main pathogenic bacteria involved in periodontitis (60, 61) (53). Therefore, consistently our data suggest that HIV-infected individuals have increased bacterial communities associated with periodontal conditions. Besides, *Prevotella* is reported to be more abundant in the gastrointestinal tract (GI) of men who have sex with men (62). The GI microbiome can be altered by different types of sexual practices. For example, men who have sex with men present a distinct GI microbiome composition when compared to men who have sex with women (63, 64). Therefore, the type of sexual practice may influence the GI and oral microbiome composition. Although we did not obtain such information from our study subjects, we had a mixed population of males/females for the microbiome analysis. Furthermore, a higher abundance of *Prevotella* is related to a lower abundance of Th17 cells and IFN-I genes expression in the GI of HIV-infected individuals (65). Moreover, the presence of *Prevotella* is associated with increased HIV acquisition in the genital tract (66). We found *Elizabethkingia* was another abundant bacteria in the saliva of HIV-infected individuals. *Elizabethkingia* is a multidrug-resistant bacteria associated with life-threatening infections in immunocompromised individuals (67). Thus, the presence of bacterial species like *Porphyromonadacea* and *Elizabethkingia* in the saliva of HIV-infected individuals may predispose them to such bacterial infections.

On the other hand, we found that *Helicobacter* was significantly decreased in the saliva of HIV-infected individuals. The presence of *Helicobacter pillory* in the dental plaque of HIV-infected individuals with *H. pillory-*induced gastritis has been reported (68, 69). However, its correlation with the stomach infection has been challenged (70) despite reports that the oral cavity should be considered as the secondary site for its colonization (71). However, our results are in agreement with another report that indicated the reduced frequency of *H. papillary*-induced gastric infection in HIV-infected individuals (72).

Our further analysis in understanding the immune components of the oral cavity in HIV-infected individuals demonstrated a significant decrease in the proportion of oral neutrophils which was associated with disease progression (e.g., CD4^+^ T cell count). Notably, LTNPs exhibited the same frequency of neutrophils in their oral cavity compared to HCs. This observation provides another novel insight into the uniqueness of this rare group of HIV-infected individuals as we have reported elsewhere (73–75). Therefore, considering the crucial role of oral neutrophils in immune homeostasis in the oral cavity, their lower frequency may predispose HIV-infected individuals to opportunistic infections. As such, it is possible to speculate that decreased frequency of oral neutrophils in individuals with lower CD4^+^ T cell count may reflect the depletion of Th17 cells at their mucosal sites (76). Subsequently, a lower Th17 cell population reduces neutrophils’ recruitment to the oral cavity. On the other hand, lower Th17 cells at the mucosal sites of the oral cavity may predispose HIV-infected individuals to oral candidiasis, considering the protective role of IL-17 against *Candida albicans* (77). Although we were unable to investigate the cross-talk between neutrophils and the oral microbiome, the salivary increase of *Treponema* species may potentially be related to neutrophil dysfunction in HIV-infected individuals (78). Deficiency in neutrophils chemotaxis and polarization in HIV-infected individuals (79) might explain another reason for reduced neutrophil frequency in HIV-infected individuals. On a supporting note, we observed significantly reduced expression and frequency of CD44^+^ neutrophils in the oral cavity of HIV-infected individuals. CD44 is expressed on neutrophils and contributes to neutrophil crawling and extravasation (35). Therefore, lower CD44 expression on neutrophils from HIV-infected individuals may provide another underlying mechanism for their impairment. Subsequently, we found elevated levels of soluble CD44 in the saliva of HIV-infected individuals compared to HCs. The role of soluble CD44 in the saliva of HIV-infected individuals is still unknown and required further investigation. However, the salivary CD44 appears to be a surrogate marker in detection of head and neck squamous cell carcinoma (HNSCC) (80). Of note, we found a positive correlation between soluble CD44 and IL-6 in the saliva of HIV-infected individuals.

IL-6 has been reported as exhibiting both pro-and anti-inflammatory functions. Certain protective aspects of IL-6 influence leukocyte migration such as prevention of neutrophil accumulation at the site of inflammation/infection (81, 82). As such, exposure of IL-6 deficient mice to respiratory endotoxin resulted in a higher number of neutrophil accumulation in their lungs compared to wild-type mice (81).

Therefore, it is possible to suggest that the saliva level of IL-6 may influence neutrophil recruitment to the oral cavity as it enhances neutrophil egress (83). On the other hand, we found that Gal-9 interaction with CD44 enhances neutrophil migration *in vitro*. However, Gal-9 appears to exhibit different effects on neutrophil chemotaxis. For example, Gal-9 deficient mice experienced reduced neutrophil response to respiratory infection (84). In contrast, increased neutrophil infiltration following ischemic injury in Gal-9 deficient mice has been reported (85). Although Gal-9 may regulate neutrophil infiltration at the site of inflammation by modulating regulatory T cells and Th17 cells (86), our observations support that Gal-9 *via* interaction with CD44 enhances neutrophil migration. Finally, the association between the saliva CD44 levels and the microbial Faith’s diversity suggests a cross-talk between neutrophils and the composition/abundance of microbial communities in the oral cavity.

We are aware of many study limitations that may have influenced our results. Although we attempted to have age-sex-matched study subjects, due to the COVID-19 pandemic we had limited options in terms of access to study cohorts. We also noted a higher prevalence of cigarette smoking, people with substance use disorder, and individual with poor oral health in the HIV-infected group, which might have influenced our results. Moreover, we were unable to analyze the impact of ART on neutrophils and/or microbiome in our cohort because all of our study subjects were on ART. Also, HIV-infected individuals had more underlying conditions compared to the HCs. However, for the microbiome studies we selected subjects without major underlying health conditions apart from HIV infection. Finally, due to a very low cell yield in the oral washes, we were unable to perform functional studies to better characterize oral neutrophil functions compared to their counterparts in the blood.

In summary, our data provide a novel insight into the impact of HIV infection on oral neutrophils. In particular, we discovered that oral neutrophils in HIV-infected individuals have significantly higher expression of CD32 but lower expression of CD44. The influence of Gal-9:CD44 on neutrophil migration highlights an important role for Gal-9 in neutrophil movement. Thus, the lower frequency of neutrophils in the oral cavity of HIV-infected individuals could be explained by the downregulation of CD44 expression. More importantly, we found elevated levels of soluble salivary CD44 which was positively correlated with Faith’s diversity of the microbiome.

Overall, our results support the differential oral microbiome diversity and richness in HIV-infected individuals. Although further studies in larger cohorts are required, our results provide a novel insight into the immune-microbiota relationship in the oral cavity.

## Data Availability Statement

The original contributions presented in the study are publiclyavailable. This data can be found here: https://www.ncbi.nlm.nih.gov/, PRJNA766045.

## Ethics Statement

The studies involving human participants were reviewed and approved by the human ethics board at the University of Alberta. The patients/participants provided their written informed consent to participate in this study.

## Author Contributions

ER performed most of the immunological and microbiome related experiments, analyzed the data and wrote the first draft. SH performed some of the experiments. JJ assisted with 16S rRNA data analysis. CO’N and ST recruited HIV-infected individuals for the study. PP provided resources and scientific advice. SE conceived the original idea, designed and supervised all of the research, assisted in data analysis and re-wrote the manuscript. All authors contributed to the article and approved the submitted version.

## Funding

This study was supported by a Foundation Grant from the Canadian Institutes of Health Research (CIHR) and a CIHR New Investigator Salary Award (both to SE).

## Conflict of Interest

The authors declare that the research was conducted in the absence of any commercial or financial relationships that could be construed as a potential conflict of interest.

## Publisher’s Note

All claims expressed in this article are solely those of the authors and do not necessarily represent those of their affiliated organizations, or those of the publisher, the editors and the reviewers. Any product that may be evaluated in this article, or claim that may be made by its manufacturer, is not guaranteed or endorsed by the publisher.
