# Supplementary material for: Differential Signature of the Microbiome and Neutrophils in the Oral Cavity of HIV-Infected Individuals
Source: Front Immunol. 2021 Nov 9;12:780910. doi: 10.3389/fimmu.2021.780910 (PMC8630784; doi:10.3389/fimmu.2021.780910)
Supplement: Supplementary file 1 [file DataSheet_1.pdf]

Supplementary Table 1. HIV-infected study subject's demographics.

Code	CD4 absolute (cells/mm3)	Viral Load (copies/mL)	Sex	Age	Experiments performed	
TH01	640	< 40	Female	26-30	Flowcytometry CD15	
TH02	590	< 40	Male	51-55	Flowcytometry CD15	ELISA, Multiplex Elisa
TH04	880	< 40	Male	51-55	Flowcytometry CD15	
TH05	730	< 40	Male	41-45	Flowcytometry CD15	
TH06	740	< 40	Male	61-65	Flowcytometry CD15	ELISA, Multiplex Elisa
TH09	560	< 40	Male	56-60	Flowcytometry CD15	
TH10	870	< 40	Male	46-50	Flowcytometry CD15	
TH11	800	< 40	Male	41-45	Flowcytometry CD15	
TH12	650	< 40	Female	41-45	Flowcytometry CD15	
TH13	630	< 40	Male	61-65	Flowcytometry CD15	
TH15	740	< 40	Female	41-45	Flowcytometry CD15	ELISA, Multiplex Elisa
TH16	550	< 40	Male	46-50	Flowcytometry CD15	ELISA, Multiplex Elisa
TH17	710	< 40	Female	31-35	Flowcytometry CD15	ELISA, Multiplex Elisa
TH18	760	< 40	Male	36-40	Flowcytometry CD15	
TH19	560	< 40	Female	36-40	Flowcytometry CD15	
TH20	570	< 40	Female	36-40	Flowcytometry CD15	
TH21	650	< 40	Male	41-45	Flowcytometry CD15	
TH170	720	< 40	Male	36-40	Flowcytometry CD15, Gal-9, CD44, CD32	
TH173	340	804	Male	31-35	Flowcytometry CD15, Gal-9, CD44, CD32	
TH175	354	< 40	Female	36-40	Flowcytometry CD15, Gal-9, CD44, CD32	
TH177	651	< 40	Female	18-25	Flowcytometry CD15, Gal-9, CD44, CD32	ELISA, Multiplex Elisa
TH178	590	< 40	Male	41-45	Flowcytometry CD15, Gal-9, CD44, CD32	ELISA, Multiplex Elisa
TH180	460	< 40	Male	46-50	Flowcytometry CD15, Gal-9, CD44, CD32	ELISA, Multiplex Elisa
TH182	302	54	Female	41-45	Flowcytometry CD15, Gal-9, CD44, CD32	ELISA, Multiplex Elisa
TH185	750	< 40	Male	41-45	Flowcytometry CD15, Gal-9, CD44, CD32	ELISA, Multiplex Elisa
TH186	590	< 40	Female	46-50	Flowcytometry CD15, Gal-9, CD44, CD32	ELISA, Multiplex Elisa
TH187	920	< 40	Male	36-40	Flowcytometry CD15, Gal-9, CD44, CD32	ELISA, Multiplex Elisa
TH189	208	314	Male	51-55	Flowcytometry CD15, Gal-9, CD44, CD32	ELISA, Multiplex Elisa
TH190	910	< 40	Female	56-60	Flowcytometry CD15, Gal-9, CD44, CD32	ELISA, Multiplex Elisa
TH191	780	51	Male	51-55	Flowcytometry CD15, Gal-9, CD44, CD32	ELISA, Multiplex Elisa
TH193	570	< 40	Female	51-55	Flowcytometry CD15, Gal-9, CD44, CD32	ELISA, Multiplex Elisa
TH194	348	< 40	Male	66-70	Flowcytometry CD15, Gal-9, CD44, CD32	ELISA, Multiplex Elisa
TH196	1200	< 40	Male	61-65	Flowcytometry CD15, Gal-9, CD44, CD32	ELISA, Multiplex Elisa
TH197	402	< 40	Male	71-75	Flowcytometry CD15, Gal-9, CD44, CD32	ELISA, Multiplex Elisa
TH198	1464	< 40	Male	56-60	Flowcytometry CD15, Gal-9, CD44, CD32	ELISA, Multiplex Elisa
TH199	539	< 40	Male	18-25	Flowcytometry CD15, Gal-9, CD44, CD32	ELISA, Multiplex Elisa
TH201	N/A	< 40	Male	26-30	Flowcytometry CD15, Gal-9, CD44, CD32	ELISA, Multiplex Elisa
TH202	539	< 40	Male	36-40	Flowcytometry CD15, Gal-9, CD44, CD32	ELISA, Multiplex Elisa
TH204	315	< 40	Male	41-45	Flowcytometry CD15, Gal-9, CD44, CD32	ELISA, Multiplex Elisa
TL01	140	< 40	Male	75+	Flowcytometry CD15	
TL03	170	< 40	Male	56-60	Flowcytometry CD15	
TL04	20	< 40	Male	41-45	Flowcytometry CD15	
TL05	140	< 40	Female	41-45	Flowcytometry CD15	
TL07	100	< 40	Female	56-60	Flowcytometry CD15	

TL08	40	< 40	Female	36-40	Flowcytometry CD15	ELISA, Multiplex Elisa
TL09	160	< 40	Male	56-60	Flowcytometry CD15	
TL171	166	< 40	Female	36-40	Flowcytometry CD15, Gal-9, CD44, CD53	
TL174	152	< 40	Male	51-55	Flowcytometry CD15, Gal-9, CD44, CD54	
TL179	161	< 40	Male	51-55	Flowcytometry CD15, Gal-9, CD44, CD55	
TL184	110	< 40	Male	51-55	Flowcytometry CD15, Gal-9, CD44, CD56	
L01	720	< 40	Male	61-65	Flowcytometry CD15	
L02	250	< 40	Female	41-45	Flowcytometry CD15	
L04	570	< 40	Female	41-45	Flowcytometry CD15	
L05	1030	< 40	Female	51-55	Flowcytometry CD15	
L06	640	< 40	Female	26-30	Flowcytometry CD15	
L07	920	< 40	Male	31-35	Flowcytometry CD15	
L08	560	< 40	Female	36-40	Flowcytometry CD15	
L09	700	< 40	Female	36-40	Flowcytometry CD15	
L10	970	< 40	Female	41-45	Flowcytometry CD15	
N01	230	N/A	Male	41-45	Flowcytometry CD15	
N02	160	N/A	Male	56-60	Flowcytometry CD15	

TH=ART patients with high CD4 (>200 cells/mm3)
TL=ART patients with low CD4 (<=200 cells/mm3)
L= Long-Term non-Progressor,
N= Naïve ART,
Samples in red were subjected to 16s Sequencing.

Supplementary Table 2. HIV-negative study subject's demographics.

Code	Sex	Age	Experiment performed	
HC1	Male	26-30	Flowcytometry CD15, Gal-9, CD44, CD32	
HC2	Male	26-30	Flowcytometry CD15, Gal-9, CD44, CD32	
HC3	Female	41-45	Flowcytometry CD15, Gal-9, CD44, CD32	
HC4	Female	41-45	Flowcytometry CD15, Gal-9, CD44, CD32	
HC5	Male	18-25	Flowcytometry CD15, Gal-9, CD44, CD32	
HC6	Female	31-35	Flowcytometry CD15, Gal-9, CD44, CD32	
HC7	Female	56-60	Flowcytometry CD15, Gal-9, CD44, CD32	
HC8	Female	46-50	Flowcytometry CD15, Gal-9, CD44, CD32	
HC9	Female	18-25	Flowcytometry CD15, Gal-9, CD44, CD32	
HC10	Female	26-30	Flowcytometry CD15, Gal-9, CD44, CD32	
HC11	Female	18-25	Flowcytometry CD15, Gal-9, CD44, CD32	
HC12	Female	26-30	Flowcytometry CD15, Gal-9, CD44, CD32	
HC13	Female	26-30		Elisa, Multiplex Elisa
HC14	Female	41-45		Elisa, Multiplex Elisa
HC15	Female	36-40		Elisa, Multiplex Elisa
HC16	Male	31-35		Elisa, Multiplex Elisa
HC17	Female	51-55		Elisa, Multiplex Elisa
HC18	Female	18-25		Elisa, Multiplex Elisa
HC19	Male	18-25		Elisa, Multiplex Elisa
HC20	Male	18-25		Elisa, Multiplex Elisa
HC21	Female	26-30	Flowcytometry CD15, Gal-9, CD44, CD32	Elisa, Multiplex Elisa
HC22	Female	26-30	Flowcytometry CD15, Gal-9, CD44, CD32	Elisa, Multiplex Elisa
HC23	Female	26-30	Flowcytometry CD15, Gal-9, CD44, CD32	Elisa, Multiplex Elisa
HC24	Male	26-30		Elisa, Multiplex Elisa
HC25	Female	51-55		Elisa, Multiplex Elisa
HC26	Female	18-25		Elisa, Multiplex Elisa
HC27	Female	18-25	Flowcytometry CD15, Gal-9, CD44, CD32	Elisa, Multiplex Elisa
HC28	Female	31-35	Flowcytometry CD15, Gal-9, CD44, CD32	Elisa, Multiplex Elisa
HC29	Male	51-55	Flowcytometry CD15, Gal-9, CD44, CD32	Elisa, Multiplex Elisa
HC30	Male	31-35	Flowcytometry CD15, Gal-9, CD44, CD32	Elisa, Multiplex Elisa
HC31	Male	31-35		Elisa, Multiplex Elisa
HC32	Male	31-35		Elisa, Multiplex Elisa
HC33	Female	18-25	Flowcytometry CD15, Gal-9, CD44, CD32	
HC34	Male	18-25	Flowcytometry CD15, Gal-9, CD44, CD32	
HC35	Female	26-30	Flowcytometry CD15	
HC36	Male	46-50	Flowcytometry CD15	
HC37	Female	36-40	Flowcytometry CD15	
HC38	Male	61-65	Flowcytometry CD15	
HC39	Male	41-45	Flowcytometry CD15	
HC40	Female	41-45	Flowcytometry CD15	
HC41	Male	26-30	Flowcytometry CD15	
HC42	Female	51-55	Flowcytometry CD15	
HC43	Female	51-56	Flowcytometry CD15	

Samples in red were subjected to 16s Sequencing.

Supplementary Table 3. Demographic information and habits.

		HC (n=43)		HIV (n=61)	
		Counts	(%)	Counts	(%)
Age	18-30	21	49	5	8
	31-40	9	21	14	23
	41-50	6	14	19	31
	51-60	6	14	16	26
	61-75	1	2	6	10
	75+	0	0	1	2
Sex	Male	16	37	37	61
	Female	27	63	24	39
Smokes	No	41	95	36	59
	Light Smoker	2	5	16	26
	Medium Smoker	0	0	4	7
	Heavy Smoker	0	0	5	8
Alcohol	Never	16	37	27	44
	Daily	3	7	3	5
	Weekly	9	21	13	21
	Monthly	15	35	18	30
Recreational substances	No use	41	95	43	70
	Cannabis	2	5	12	20
	Others (oxycodone, crack, cocaine, crystal meth)	0	0	1	2
	Cannabis+ others	0	0	5	8
Medication	Only ART	0	0	37	60
	Antibiotics+ ART	0	0	6	10
	Other	13	30	18	30
	None	30	70	0	0
