# Supplementary material for: Differential Signature of the Microbiome and Neutrophils in the Oral Cavity of HIV-Infected Individuals
Source: Front Immunol. 2021 Nov 9;12:780910. doi: 10.3389/fimmu.2021.780910 (PMC8630784; doi:10.3389/fimmu.2021.780910)
Supplement: Supplementary file 2 [file DataSheet_2.pdf]

Supplementary Figure 1

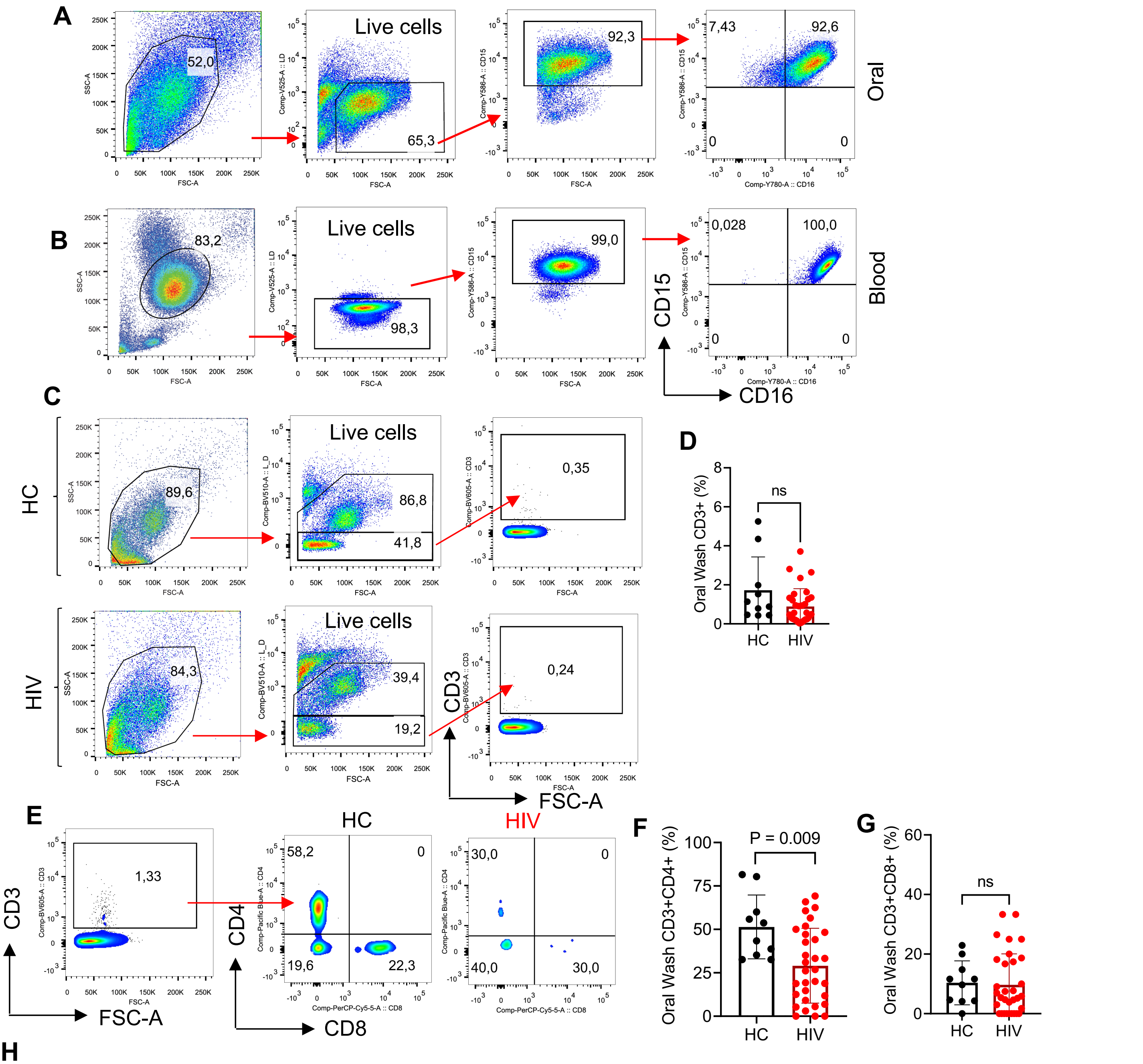

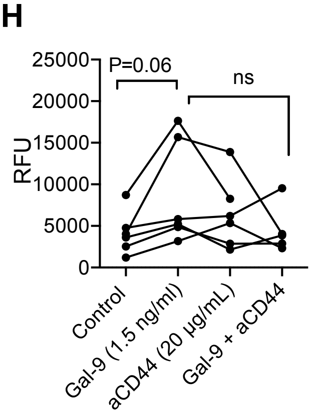

S. Figure 1. (**A**) Representative flow cytometry plots showing the gating strategy and the purity of oral neutrophils obtained from the oral wash. (**B**) Representative flow cytometry plots showing the gating strategy and the purity of blood neutrophils. (**C**) Representative gating strategy, and (**D**) cumulative data for $CD3^{+}$ T cells in the oral wash. (**E**) Representative plots, and (**F**) cumulative data for $CD4^{+}$, and (**G**) $CD8^{+}$ T cells in the oral wash of HCs and HIV-infected individuals. (**H**) Cumulative data showing oral neutrophil migration in the presence of soluble Gal-9 (1.5 ng/ml), anti-CD44 antibody (20 μg/ml) or soluble Gal-9 (1.5 ng/ml) plus anti-CD44 antibody while all well were treated with fMLP (50 μm).
